# Etiology and clinical characteristics of pediatric non-neoplastic posterior reversible encephalopathy: systematic review

**DOI:** 10.1097/j.pbj.0000000000000147

**Published:** 2022-06-17

**Authors:** Mariana Jordão, Pedro Raimundo, Ruben Jacinto, Daniel Garrido, João Martins, Rui Estevens, Jerina Nogueira, Emanuel Fernandes, Ana Macedo, Hipólito Nzwalo

**Affiliations:** a Faculty of Medicine and Biomedical Sciences of Algarve, Algarve,; b Internal Medicine Department, Hospital Dr. José Maria Grande, Portalegre,; c Algarve Biomedical Center,; d Stroke Unit, Centro Hospitalar Universitário do Algarve, Algarve, Portugal

**Keywords:** “posterior reversible encephalopathy syndrome”, children, etiology, non-neoplastic

## Abstract

Although more commonly seen in adult population, posterior reversible encephalopathy syndrome (PRES) can also be observed in pediatric patients. The etiopathogenesis of pediatric PRES is poorly understood, and the available evidence comes mostly from childhood cancer. Analysis of the sociodemographic and clinical characteristics of the different noncancer-related types can improve the understanding of pediatric PRES.

**Methods:**

Systematic review of characteristics and outcome of noncancer pediatric PRES. Primary sources of investigation were identified and selected through Pubmed and Scopus databases. The research was complemented by reference search in relevant publications. Study protocol was registered (Prospero CRD42020165798).

**Results:**

We identified 449 cases of noncancer pediatric PRES from 272 studies, median age 10 (newborn to 17 years), 49.9% girls. The 4 most common groups of conditions were renal 165 (36.7%), hematologic 84 (18.7%), autoimmune 64 (14.3%), and cardiovascular 28 (6.2%) disorders. The 4 most prevalent precipitants identified were hypertensive crisis 119 (26.5%), corticosteroids 56 (12.5%), immunosuppression drugs 44 (9.8%), and biologic drugs 14 (3.1%). Clinical presentations included seizures 100 (22.3%), headaches 22 (4.9%), encephalopathy 17 (3.8%), visual disturbances 6 (1.3%), and focal deficit 3 (0.7%). The distribution of lesions was (n = 380): combined anterior/posterior circulation (40.8%), isolated posterior circulation (24.1%), anterior circulation (6.2%), and deep structures (1.6%). Residual neurological deficits occurred in about 1 out 10 cases.

**Conclusion:**

Pediatric PRES differs from the adult in etiology, precipitants, and clinical manifestations. Renal diseases predominate, acute raised blood pressure is less frequent, and cortical deficits are rarer. In addition, the proportion of patients with combined anterior/posterior circulation was higher. Permanent neurological sequels can occur.

## Introduction

Although rare, posterior reversible encephalopathy syndrome (PRES) is becoming increasingly recognized in pediatrics.^1^ Childhood PRES presents with a variety of combined central nervous system manifestations namely headache, seizures, altered level of consciousness, behavior changes, cortical, and pyramidal signs.^2–4^ Typically, there is vasogenic edema involving posterior cerebral regions.^2–4^ Although reversible by definition, poor neurological functional outcome is a possibility in childhood PRES.^5^ For instance, in critically ill pediatric patients, when matched by age and severity of the underlying disease, children with PRES have higher mortality risk.^6^ The pathophysiology of PRES remains unclear, but endothelial lesion with disruption of the blood-brain barrier or exhaustion of the vascular autor-egulatory mechanisms leading to fluid and protein transudation in the brain are the principal hypothesis.^6^ There are previously published reviews of pediatric PRES. These reviews, however, were focused on very specific diseases or conditions,^2–4^ and did not address systematically the sociodemographic, clinical, and radiological evolution.^7^ Comparison of the etiologies and clinical and radiological manifestations of pediatric PRES can point toward the mechanisms and help clarifying the factors that might contribute to the prognosis. Thus, we reviewed the etiology, pathophysiological, clinical, and radiological aspects of non-oncology-related PRES in children.

## Methods

Pubmed and Scopus databases were used to search for relevant publications up to February 28, 2019 using the combination of the following MeSH terms: “posterior leukoencephalopathy syndrome” [MeSH Terms] OR (“posterior”[All Fields] AND “leucoencephalopathy”[All Fields] AND “syndrome”[All Fields]) OR “posterior leukoencephalopathy syndrome”[All Fields] OR (“posterior”[All Fields] AND “reversible”[All Field] AND “encephalopathy”[All Fields]) OR “posterior reversible encephalopathy syndrome” [All Fields]. This search was complemented by examining reference lists of the most relevant studies. Studies or case reports containing the description of clinical cases of childhood PRES; with available information about precipitants,sociodemographic,and clinical characteristics were included. We excluded article based on cancer-related PRES to overcome the possible limitation of prognosis or outcome being influenced by the disease itself not the occurrence of PRES. Cases without brain imaging confirmation or minimal etiological investigation (inflammatory, renal, cardiovascular, and hemato-logical) were not included. Non-English studies were also excluded. All obtained titles and abstracts were independently verified by 2 investigators. Disagreements regarding the inclusion of specific studies were resolved by a third investigator. This study was based mainly on information extracted from single case reports and therefore quality evaluation was not performed. The systematic review was registered and accepted at PROSPERO (CRD42020165798).

## Results

### Data collection

**Figure 1.**
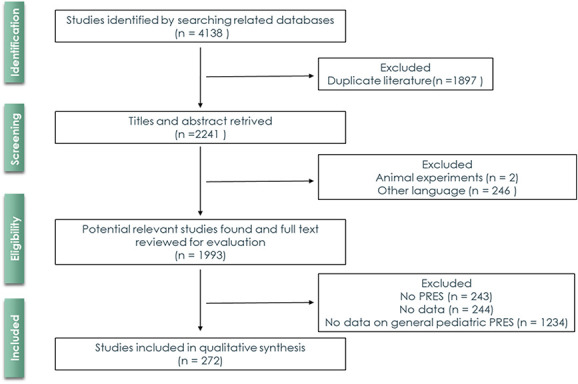
Prisma flowchart process of selection and inclusion of articles in the systematic review.

A total of 4138 references were initially retrieved. After automatic removal of duplicated manuscripts, title and abstract of 2241 articles were screened. Preferred Reporting Items for Systematic Reviews and Meta-Analyses flowchart diagram (Fig. 1) resumes the selection and inclusion process. A total of 1993 articles were selected for complete text evaluation, after which, 272 studies were included. The total number of patients included was 449 (Table 1 in the supplement).

**Table 1:** Distribution of symptoms among to the 4 main group of etiologies of pediatric non-neoplastic posterior reversible encephalopathy syndrome

	Symptoms				
Etiology	Seizures (%)	Headache (%)	Visual disturbances (%)	Encephalopathy (%)	Focal deficit (%)
Autoimmune	81.3	64.1	29.7	17.2	9.4
Cardiovascular	85.7	53.6	25.0	17.9	3.6
Hematologic	65.6	50.1	33.4	27.6	9.6
Renal	79.3	52.1	36.9	30.2	9.0

The main reasons for exclusion were cancer-related pediatric PRES (1234/55.1%), non-English publications (246/11%), inexistence of data (244/10.9%), non-PRES papers (243/10.8%), and animal experiments (2/0.09%). The overall median age was 10 (range: newborn to 17years), with almost no sex difference (girls = 224/49.9%). The 4 main etiologies were identified: renal (165/36.7%), hematological (84/18.7%), autoimmune (64/14.3%), and cardiovascular (28/6.2%).Overall, the most common precipitants of PRES were acute raised blood pressure (119/26.5%), corticoids (56/12.5%), immunosuppression (44/9.7%), acute inflammation (16/3.6%), and biologic drugs (14/3.1%).

### Etiologies

#### Autoimmune

Sixty-four cases of autoimmune PRES were identified. The average age was 12.7 ± 3.9years, with female predominance (64.1%). Several etiologies were considered, among which the most representative were systemic lupus erythematosus (62.5%), Guillan-Barret syndrome (9.4%), Takayasu arteritis (6.3%), juvenile idiopathic arthritis (4.7%), and autoimmune hemolytic anemia (3.3%). At the clinical presentation, the most common symptoms were seizures, headache, and visual disturbances (Table 1). The majority (44/68.8%) of patients had onset of systolic blood pressure greater than 140 mm Hg. PRES involved the posterior circulation predominantly (70.3%). Coexisting anterior circulation lesions was common (45.3%). Only 2 children had isolated lesions in the deep brain structures.

#### Cardiovascular

In the cardiovascular disease group, there were a total of 28 patients, with an average age of 10.5±3.9years, without gender disbalance (boys = 53.6%). The most representative etiologies were hypertension (78.6%) and dilated cardiomy-opathy (7.1%). The most common presenting symptoms were seizures, headache, and visual disturbances (Table 1). At onset, most children had systolic blood pressure greater than 140 mm Hg (78.6%). Posterior circulation lesions were common (71.4%), with coexistent anterior circulation occurring in 60.7% of them. No child had isolated lesions in the deep structures.

#### Hematologic

A total of 84 patients, average age of 9.4 ± 3.9 years, 50% girls, had PRES in the context of hematological conditions. The most representative etiologies were sickle cell anemia (23.8%), Henoch-Schönlein purpura (16.7%), β-thalas-semia (14.3%), and hemolytic uremic syndrome (10.7%). Frequent manifestations were seizures, headache, and visual disturbances (Table 1). Systolic blood pressure above 140mm Hg was documented in n = 56 (66.7%). In terms of imaging, most of the patients had radiological findings in the posterior circulation (69%), with concomitant involvement of the anterior circulation 48.8%. One child had isolated lesions in the deep brain structures.

#### Renal

PRES occurred in 165 patients with underlying kidney disease. The average age was 10.1 ± 4years and 52.1% were boys. Of the varied list of etiologies, the most representative were nephrotic syndrome (26.7%), chronic kidney disease (12.7%), post-streptococcal glomerulonephritis (12.1%), and glomerulonephritis (10.9%). At the beginning of the clinical presentation, common symptoms were seizures, headache, and visual disturbances (Table 1). Acute blood systolic pressure above 140mm Hg was common (117/70.9%). In terms of imaging, most patients had posterior circulation (77.6%) lesions; of these, 50.3% also exhibited anterior circulation lesions. Isolated deep structures located PRES occurred in a single child.

#### Precipitants

The 4 most prevalent precipitants were hypertensive crisis n = 119 (26.5%), corticosteroids n = 56 (12.5%), immunosuppression drugs (cyclosporine, azathioprine, tacrolimus, others) n = 44 (9.8%), and biologic drugs (basiliximab, infliximab, eculizumab, others) n = 14 (3.1%).

Table 2 shows the most common diseases for each precipitant. Patients identified with hypertensive crisis as the precipitant, the most common underlying diseases were essential hypertension (n = 19; 16.0%), post-streptococcal glomerulonephritis (n = 13; 10.9%), chronic kidney disease (n = 12; 10.1%), sickle cell anemia (n = 8; 6.7%), nephrotic syndrome (n = 8; 6.7%), and systemic lupus erythematosus (n = 6; 5.0%). Corticosteroids were identified as precipitant of PRES in underlying diseases such as systemic lupus erythematosus (n = 12; 21.4%), nephrotic syndrome (n = 12; 21.4%), kidney transplant (n = 6; 10.7%), and Henoch Schönlein Purpura (n = 4; 7.1%). Immunosuppression drugs were identified as precipitant of PRES in nephrotic syndrome (n = 12; 27.3%), β-talassemia (n = 6; 13.6%), kidney transplant (n = 6; 13.6%), and glomerulonephritis (n = 3; 6.8%). Biologic drugs were found as a precipitant of PRES in aplastic anemia (n = 2; 14.3%), Crohn disease (n = 2; 14.3%), nephrotic syndrome (n = 2; 14.3%), and obliterans bronchitis (n = 2; 14.3%).

**Table 2:** Most common diseases and correspondent precipitant of non-neoplastic pediatric posterior reversible encephalopathy syndrome

Precipitant	Most common underlying disease—n (%)
Hypertensive crisis	Hypertension (n = 19; 16.0%) Post-streptococcal glomerulonephritis (n = 13; 10.9%) Chronic kidney disease (n = 12; 0.1%) Sickle cell anemia (n = 8; 6.7%) Nephrotic syndrome (n =8; 6.7%) Systemic lupus erythematosus (n = 6; 5.0%) Other diseases (n = 53; 44.6%)
Corticosteroids	Systemic lupus erythematosus (n = 12; 21.4%) Nephrotic syndrome (n =12; 21.4%) Kidney transplant (n =6; 10.7%) Henoch Schönlein purpura (n =4; 7.1%) Other diseases (n= 22; 39.4%)
Immunosuppression drugs	Nephrotic syndrome (n =12; 27.3%) β-Thalassemia (n= 6; 13.6%) Kidney transplant (n =6; 13.6%) Glomerulonephritis (n =3; 6.8%) Other diseases (n= 17; 38.7%)
Biologic drugs	Aplastic anemia (n =2; 14.3%) Crohn disease (n= 2; 14.3%) Nephrotic syndrome (n =2; 14.3%) Obliterans bronchitis (n = 2; 14.3%) Other diseases (n= 6; 42.8%)

#### Prognosis

**Figure 2.**
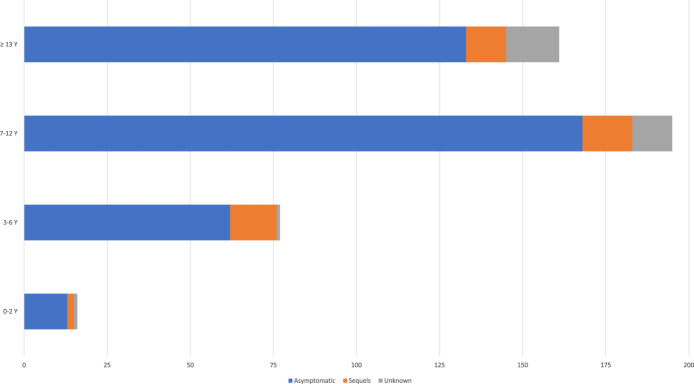
Proportion of patients with residual neurological deficits after pediatric non-neoplastic posterior reversible encephalopathy syndrome.

**Figure 3.**
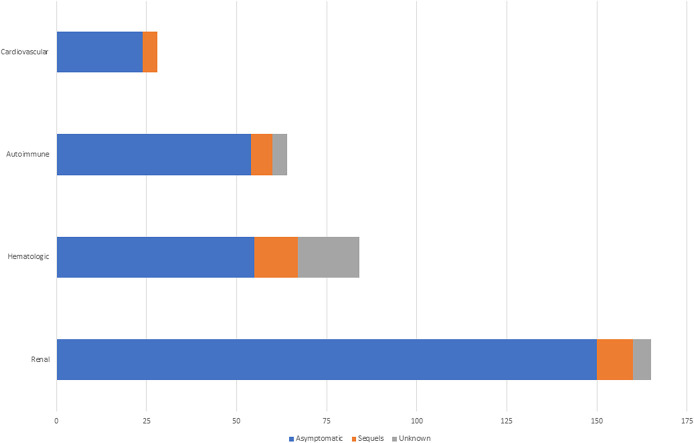
Proportion of patients with residual neurological deficits after pediatric non-neoplastic posterior reversible encephalopathy syndrome.

The majority (376/83.7%) of children were asymptomatic after resolution of PRES, and n = 43 (9.6%) had sequels defined as any residual neurological deficit on the follow-up. The proportion of patients with residual neurological deficits did not differ between age groups (Fig. 2). Figure 3 depicts the proportion of patients showing favorable outcome after imaging resolution of PRES based on the etiology. Of the 449 cases analyzed, intracerebral hemorrhage occurred in 4 (0.9%) children: 2 with sickle cell anemia, 1 with heart transplant, and 1 with nephrotic syndrome. In 105 cases (23.4%) no information of follow up imaging was retrieved. The age of intracranial hemorrhage group ranged from 3 to 10years old, the mean age was 6.25years.

## Discussion

The great majority of our patients had ages ranging from 7 to 12 years, whereas in other systematic reviews, the mean age presentation is more commonly found in adolescent age groups (≥12years old).^8^ Although the exact incidence of PRES in children is not known, it is estimated to be rare.^8,9^

In comparison to PRES associated with cancer, the proportion of children with less than 10 years was higher.^8^ It is reasonable to speculate that in PRES associated with cancer, endothelial dysfunction, not failure of autoregulation may play a significant role. If that is the case, the immature brain, more susceptible to the toxic effects of chemotherapy may suffer more. We did not find sex differences in the occurrence of pediatric PRES. In adults, the slightly higher predominance in female population compared to male is still to be clarified.^10^ Etiological aspects may account for this finding, since women are more prone to acute inflammation disorders,^11^ which are more frequent in adults as compared to children (45% on average in adults vs 14.3% in our review).^12^ It is hypothesized that children have a narrower range of cerebral autoregulation and therefore the probability of having PRES is even higher than that in adults, in situations such as acute rise in blood pressure.^13,14^ Prompt recognition and appropriate management potentially improves the diagnosis.^15^ Our systematic review has shown that the proportion of patients with cortical signs, in particular visual signs,^16^ which occurs in a third of adult PRES, is lower in children. This finding however may reflect reporting bias, because very young children may have difficulties in complaining about visual symptoms. Indeed, visual disturbances were reported in 29.7% of the children from the autoimmune diseases group, composed by older patients. These symptoms are typically in PRES presentation^17^; however, the presentation of these grouped symptoms was higher than its presentation in isolation. Consistent with the findings from the majority of studies, our systematic review showed that regardless of the etiology of PRES, seizures emerged as the main manifestation.^17,18^ However, we found some differences in the distribution of the main clinical manifestations of pediatric PRES potentially reflecting the characteristics of the underlying etiology. For instance, the frequency of encephalopathy in renal diseases and headache in autoimmune diseases was higher, and can be attributed to uremia toxicity and systemic inflammation, respectively. We found that 65.3% of children had a systolic pressure greater than 140 mm Hg when PRES was detected which is in favor of the hyperperfusion hypothesis of PRES. On the contrary, the elevated proportion of nonhypertension-related PRES suggests that endothelial dysfunction may be more important in children. The immature brain vessels may be more susceptible to the different exogenous and endogenous circulatory toxins, which when in contact with the cerebral endothelium, damage the blood-brain barrier, allowing the extravasation of fluid, and plasma components.^19^ Contrary to the findings from adult PRES, where the frequency of combined anterior and posterior circulation rarely exceeds 30%,^16^ in our systematic review, the majority of childhood had combined anterior and posterior brain involvement. Whether this suggest a more vulnerable brain or different mechanisms are the basis of PRES should be clarified and may contribute to the etiopathogenesis.

There are some limitations worth to be highlighted. By excluding non-English publications from our evidence synthesis the risk of language bias is to be considered. In addition, publication bias favoring publication of dramatic or well succeed cases is also a possibility.

PRES should always be considered in differential diagnosis with any neurological symptom.^20^ Although reversible in the majority, permanent sequels can occur.^21^ In conclusion, our systematic review has shown pediatric non-cancer related PRES has very particular characteristics. Different from adult PRES where hypertension predominate, in children, kidney diseases pay an important etiological role. From clinical standpoint of view, cortical deficits, particularly visual changes, are less frequent in childhood noncancer-related PRES. In addition, in comparison to adults, the proportion of patients with combined anterior/posterior circulation is higher. Together, these findings suggest that in childhood PRES the contribution endothelial toxicity is the main factor involved.
